# Supplementary material for: Assessment of Saudi Mothers’ Attitudes towards Their Children’s Pain and Its Management
Source: Int J Environ Res Public Health. 2021 Jan 5;18(1):348. doi: 10.3390/ijerph18010348 (PMC7796466; doi:10.3390/ijerph18010348)
Supplement: Supplementary file 1 [file ijerph-18-00348-s001.pdf]

International Journal of
Environmental Research and Public Health

MDPI

Table 1. Association between attitude items and mothers' education level and age.

Items	Demographic variables of mothers	Disagree *n* (%)	Unsure *n* (%)	Agree *n* (%)	*P* value
Q1	Education level				
	Primary / Secondary School	5(23.8)	1(4.8)	15(71.4)	
	High School	2(4.9)	5(12.2)	34(82.9)	0.023
	University	12(4.2)	38(13.2)	238(82.6)	
	Post Graduate	3(6.1)	7(14.3)	39(79.6)	
	Age group				
	25–35 years	9(4.0)	28(12.4)	188(83.6)	
	36–45 years	8(6.7)	18(15.1)	93(78.2)	0.410
	46–55years	5(9.6)	4(7.7)	43(82.7)	
	More than 55	0	1(33.3)	2(66.7)	
Q2	Education Level				
	Primary / Secondary School	6(28.6)	7(33.3)	8(38.1)	
	High School	6(14.6)	18(43.9)	17(41.5)	0.616
	University	45(15.6)	120(41.7)	123(42.7)	
	Post Graduate	8(16.3)	25(51.0)	16(32.7)	
	Age group				
	25–35 years	38(16.9)	98(43.6)	89(39.6)	
	36–45 years	15(12.6)	52(43.7)	52(43.7)	0.589
	46–55years	12(23.1)	18(34.6)	22(42.3)	
	More than 55	0	2(66.7)	1(33.3)	
Q3	Education Level				
	Primary / Secondary School	9(42.9)	9(42.9)	3(14.3)	
	High School	15(36.6)	19(46.3)	7(17.1)	0.573
	University	130(45.1)	106(36.8)	52(18.1)	
	Post Graduate				
	Age group				
	25–35 years	103(45.8)	79(35.1)	43(19.1)	
	36–45 years	55(46.2)	45(37.8)	19(16.0)	0.819
	46–55years	22(42.3)	23(44.2)	7(13.5)	
	More than 55	2(66.7)	1(33.3)	0	
Q4	Education Level				
	Primary / Secondary School	2(9.5)	11(52.4)	8(38.1)	
	High School	4(9.8)	17(41.5)	20(48.8)	0.443
	University	34(11.8)	96(33.3)	158(54.9)	
	Post Graduate	9(18.4)	16(32.7)	24(49.0)	
	Age group				
	25–35 years	29(12.9)	86(38.2)	110(48.9)	
	36–45 years	14(11.8)	34(28.6)	71(59.7)	0.620
	46–55years	6(11.5)	19(36.5)	27(51.9)	
	More than 55	0	1(33.3)	2(66.7)	
Q5	Education Level				
	Primary / Secondary School	4(19.0)	6(28.6)	11(52.4)	
	High School	4(9.8)	16(39.0)	21(51.2)	0.545
	University	21(7.3)	95(33.0)	172(59.7)	
	Post Graduate	3(6.1)	18(36.7)	28(57.1)	
	Age group				
	25–35 years	15(6.7)	93(41.3)	117(52.0)	
	36–45 years	14(11.8)	28(23.5)	77(64.7)	0.012
	46–55years	3(5.8)	14(26.9)	35(67.3)	
	More than 55	0	0	3(100)	
Q6	Education Level				
	Primary / Secondary School	3(14.3)	5(23.8)	13(61.9)	
	High School	11(26.8)	8(19.5)	22(53.7)	0.202
	University	65(22.6)	99(34.4)	124(43.1)	
	Post Graduate	15(30.6)	17(34.7)	17(34.7)	
	Age group				
	25–35 years	56(24.9)	73(32.4)	96(42.7)	0.858
	36–45 years	28(23.5)	40(33.6)	51(42.9)	
	46–55years	10(19.2)	15(28.8)	27(51.9)	

	More than 55	0	1(33.3)	2(66.7)	
Q7	Education Level				
	Primary / Secondary School	12(57.1)	4(19.0)	5(23.8)	
	High School	18(43.9)	19(46.3)	4(9.8)	0.344
	University	146(50.7)	93(32.3)	49(17.0)	
	Post Graduate	24(49.0)	19(38.8)	6(12.2)	
	Age group				
	25–35 years	108(48.0)	83(36.9)	34(15.1)	
	36–45 years	63(52.9)	33(27.7)	23(19.3)	0.664
	46–55years	27(51.9)	18(34.6)	7(13.5)	
	More than 55	2(66.7)	1(33.3)	0	
Q8	Education Level				
	Primary / Secondary School	6(28.6)	7(33.3)	8(38.1)	
	High School	13(31.7)	11(26.8)	17(41.5)	0.331
	University	83(28.8)	126(43.8)	79(27.4)	
	Post Graduate	12(24.5)	24(49.0)	13(26.5)	
	Age group				
	25–35 years	68(30.2)	91(40.4)	66(29.3)	
	36–45 years	33(27.7)	49(41.2)	37(31.1)	0.801
	46–55years	12(23.1)	26(50.0)	14(26.9)	
	More than 55	1(33.3)	2(66.7)	0	
Q9	Education Level				
	Primary / Secondary School	5(23.8)	9(42.9)	7(33.3)	
	High School	14(34.1)	20(48.8)	7(17.1)	0.424
	University	110(38.2	108(37.5)	70(24.3)	
	Post Graduate	13(26.5)	22(44.9)	14(28.6)	
	Age group				
	25–35 years	78(34.7)	90(40.0)	57(25.3)	
	36–45 years	43(36.1)	46(38.7)	30(25.2)	0.929
	46–55years	19(36.5)	22(42.3)	11(21.2)	
	More than 55	2(66.7)	1(33.3)	0	
Q10	Education Level				
	Primary / Secondary School	8(38.1)	7(33.3)	6(28.6)	
	High School	15(36.6)	17(41.5)	9(22.0)	0.433
	University	85(29.5)	91(31.6)	112(38.9)	
	Post Graduate	18(36.7)	15(30.6)	16(32.7)	
	Age group				
	25–35 years	80(35.6)	81(36.0)	64(28.4)	
	36–45 years	37(31.1)	32(26.9)	50(42.0)	0.005
	46–55years	8(15.4)	17(32.7)	27(51.9)	
	More than 55	1(33.3)	0	2(66.7)	
Q11	Education Level				
	Primary / Secondary School	6(28.6)	9(42.9)	6(28.6)	
	High School	14(34.1)	19(46.3)	8(19.5)	0.760
	University	87(30.2)	116(40.3)	85(29.5)	
	Post Graduate	12(24.5)	19(38.8)	18(36.7)	
	Age group				
	25–35 years	67(29.8)	96(42.7)	62(27.6)	
	36–45 years	39(32.8)	43(36.1)	37(31.1)	0.870
	46–55years	12(23.1)	23(44.2)	17(32.7)	
	More than 55	1(33.3)	1(33.3)	1(33.3)	
Q12	Education Level				
	Primary / Secondary School	4(19.0)	4(19.0)	13(61.9)	
	High School	8(19.5)	16(39.0)	17(41.5)	0.385
	University	62(21.5)	118(41.0)	108(37.5)	
	Post Graduate	13(26.5)	19(38.8)	17(34.7)	
	Age group				
	25–35 years	46(20.4)	96(42.7)	83(36.9)	
	36–45 years	29(24.4)	41(34.5)	49(41.2)	0.363
	46–55years	10(19.2)	20(38.5)	22(42.3)	
	More than 55	2(66.7)	0	1(33.3)	
Q13	Education Level				
	Primary / Secondary School	3(14.3)	7(33.3)	11(52.4)	0.480
	High School	1(2.4)	16(39.0)	24(58.5)	

	University	43(14.9)	100(34.7)	145(50.3)	
	Post Graduate	8(16.3)	19(38.8)	22(44.9)	
	Age group				
	25–35 years	22(9.8)	88(39.1)	115(51.1)	
	36–45 years	24(20.2)	40(33.6)	55(46.2)	0.010
	46–55years	7(13.5)	14(26.9)	31(59.6)	
	More than 55	2(66.7)	0	1(33.3)	
	Education Level				
	Primary / Secondary School	5(23.8)	4(19.0)	12(57.1)	
Q14	High School	4(9.8)	15(36.6)	22(53.7)	0.638
	University	53(18.4)	76(26.4)	159(55.2)	
	Post Graduate	7(14.3)	14(28.6)	28(57.1)	
	Age group				
	25–35 years	43(19.1)	70(31.1)	112(49.8)	
	36–45 years	18(15.1)	26(21.8)	75(63.0)	0.232
	46–55years	7(13.5)	13(25.0)	32(61.5)	
	More than 55	1(33.3)	0	2(66.7)	
